# Myeloid-derived suppressor cell function and epigenetic expression evolves over time after surgical sepsis

**DOI:** 10.1186/s13054-019-2628-x

**Published:** 2019-11-13

**Authors:** McKenzie K. Hollen, Julie A. Stortz, Dijoia Darden, Marvin L. Dirain, Dina C. Nacionales, Russell B. Hawkins, Michael C. Cox, Maria-Cecilia Lopez, Jaimar C. Rincon, Ricardo Ungaro, Zhongkai Wang, Quran Wu, Babette Brumback, Marie-Pierre L. Gauthier, Michael Kladde, Christiaan Leeuwenburgh, Mark Segal, Azra Bihorac, Scott Brakenridge, Frederick A. Moore, Henry V. Baker, Alicia M. Mohr, Lyle L. Moldawer, Philip A. Efron

**Affiliations:** 1 Department of Surgery, Shands Hospital, University of Florida College of Medicine, Room 6116, 1600 SW Archer Road, Gainesville, FL 32610-0019 USA; 2 Department of Molecular Genetics and Microbiology, University of Florida College of Medicine, Gainesville, FL USA; 3 Department of Biostatistics, University of Florida College of Medicine, Gainesville, FL USA; 4 Department of Biochemistry and Molecular Biology, University of Florida College of Medicine, Gainesville, FL USA; 5 Department of Aging and Geriatric Research, University of Florida College of Medicine, Gainesville, FL USA; 6 Department of Medicine, University of Florida College of Medicine, Gainesville, FL USA

**Keywords:** Myeloid-derived suppressor cells, Sepsis, Surgery, Human, Epigenetics, miRNA, Immunosuppression

## Abstract

**Background:**

Sepsis is an increasingly significant challenge throughout the world as one of the major causes of patient morbidity and mortality. Central to the host immunologic response to sepsis is the increase in circulating myeloid-derived suppressor cells (MDSCs), which have been demonstrated to be present and independently associated with poor long-term clinical outcomes. MDSCs are plastic cells and potentially modifiable, particularly through epigenetic interventions. The objective of this study was to determine how the suppressive phenotype of MDSCs evolves after sepsis in surgical ICU patients, as well as to identify epigenetic differences in MDSCs that may explain these changes.

**Methods:**

Circulating MDSCs from 267 survivors of surgical sepsis were phenotyped at various intervals over 6 weeks, and highly enriched MDSCs from 23 of these samples were co-cultured with CD3/CD28-stimulated autologous T cells. microRNA expression from enriched MDSCs was also identified.

**Results:**

We observed that MDSC numbers remain significantly elevated in hospitalized sepsis survivors for at least 6 weeks after their infection. However, only MDSCs obtained at and beyond 14 days post-sepsis significantly suppressed T lymphocyte proliferation and IL-2 production. These same MDSCs displayed unique epigenetic (miRNA) expression patterns compared to earlier time points.

**Conclusions:**

We conclude that in sepsis survivors, immature myeloid cell numbers are increased but the immune suppressive function specific to MDSCs develops over time, and this is associated with a specific epigenome. These findings may explain the chronic and persistent immune suppression seen in these subjects.

## Background

Sepsis is defined as a life-threatening organ dysfunction induced by a dysregulated host response to infection, and sepsis remains one of the leading causes of preventable deaths in hospitals [1, 2]. The Agency for Healthcare Research and Quality (AHRQ) recently ranked sepsis as the most expensive condition treated in US hospitals, with estimated costs exceeding $24 billion dollars annually [3, 4]. The enormity of the problem has been recognized by the World Health Organization (WHO), which made sepsis a global health priority in 2017 [5].

Due in large part to the improvements in sepsis recognition and in subsequent treatment in intensive care units (ICUs), less than 10% of surgical sepsis patients (who can be adequately resuscitated) now die within the first 14 days of their hospital admission [6]. Although over half of surgical sepsis survivors rapidly recover and are discharged from the ICU within 14 days, nearly 50% of them (or ~ 1/3 of all surgical sepsis patients) develop what has been described as *chronic critical illness* (CCI). CCI is defined as patients who have prolonged stays in the ICU with unresolved organ dysfunction [7]. CCI can manifest itself as a persistent inflammation, immunosuppression, and catabolism syndrome (PICS) [7–11], which is associated with dismal long-term outcomes, including poor cognitive and physical function, as well as self-reported quality of life [6, 12]. CCI patients extensively utilize resources as well as accrue significant personal and hospital financial burdens [13].

To date, immune modulation therapy and pharmacotherapeutic agents have proven disappointing in amending septic patient outcomes [14], although some novel agents have demonstrated potential for beneficially moderating septic patients’ immune responses [15, 16]. There are no specific treatments for sepsis survivors who experience CCI, due in part to an inadequate knowledge of its pathobiology [17, 18]. However, we have hypothesized that the persistent low-grade inflammation in PICS patients induces a *myelodyscrasia*, which includes increased bone marrow and extramedullary proliferation of myeloid-derived suppressor cells (MDSCs) [13, 19].

MDSCs are a heterogeneous group of immature myeloid cells first discovered in cancer patients but found to be important in many disease processes including sepsis [7, 20]. Although MDSCs have several activities, the sine qua non or required ability for a cell to be classified as a MDSC is its ability to suppress lymphocyte proliferation [11, 20]. In sepsis, MDSCs are thought to be beneficial when *acutely* recruited to inflamed tissues [21], as MDSCs are capable of suppressing acute inflammatory responses and resolving inflammation [22–24]. However, if this MDSC expansion and infiltration perpetuates, the long-term persistence of MDSCs can induce significant pathophysiology leading to CCI and subsequently PICS [22, 23]. This includes host immunosuppression, an established post-septic pathology that contributes to worsened septic patient outcomes [21, 25]. In murine sepsis models, MDSCs have been found to expand in secondary lymphoid organs within 5 days and to persist for at least 12 weeks with the MDSCs inhibiting T cell proliferation via iNOS and arginase 1 production in part [26–28]. In human patients, the proportion of the different subsets of MDSCs are noted to expand differently depending on the microbial origin of sepsis [29–31].

MDSCs are also known to be phenotypically labile cells, capable of changing as well as undergoing terminal differentiation [32, 33]. Thus, MDSCs are a promising cell for immunomodulation therapies [32, 33]. However, the function and characterization of these cells in human sepsis remains undefined. Important to cellular transcriptional/epigenetic modification are microRNAs (miRs). miRNAs are a class of small, non-coding RNAs that regulate gene expression involved in cell development and differentiation. Altered miR expression affects the expansion of immature myeloid cell populations [34]. miRs function at the molecular level and can target proteins that are involved in myeloid lineage differentiation and maturation; thus, they represent a potential MDSC therapeutic target that can be readily manipulated [34].

In murine sepsis, leukocytes that meet the defined cell surface phenotype for MDSCs have a varying functionality depending on the time point from which these cells are isolated after the septic insult [35]. The phenotypic plasticity of these cells over time after human sepsis remains undefined, and a better understanding of MDSC function after the onset of human sepsis is required to successfully apply precision medicine to these patients. While the pathophysiology of sepsis remains highly complex, we examined whether the function of MDSCs evolves over time after sepsis in survivors who develop CCI. We also asked whether changes in the miR expression patterns over time in these sepsis survivors parallel change in MDSC function and phenotype.

## Methods

### Study site and patients

Over the 4-year period during which the study was conducted, 365 surgical intensive care unit (ICU) patients were enrolled who were either admitted with or subsequently developed sepsis during their hospitalization [36]. Screening for sepsis was carried out using the Modified Early Warning Signs-Sepsis Recognition System (MEWS-SRS), which quantifies derangements in vital signs, white blood cell count, and mental status [37]. All patients with sepsis were managed using a standardized, evidence-based protocol that emphasizes early goal-directed fluid resuscitation as well as other time-appropriate interventions such as administration of broad-spectrum antibiotics. Empiric antibiotics were chosen based on current hospital antibiograms in conjunction with the suspected source of infection [38]. Antimicrobial therapy was then narrowed based on culture and sensitivity data. If a patient did not improve on this standardized empiric antibiotic regimen, a consult was placed to infectious disease for alternative recommendations.

### Inclusion and exclusion criteria

Patients eligible for participation in the study met the following inclusion criteria: (1) admission to the surgical or trauma ICU; (2) age ≥ 18 years; (3) clinical diagnosis of sepsis, severe sepsis, or septic shock with this being the patient’s first septic episode; and (4) entrance into our sepsis clinical management protocol [36].

Patients were excluded if any of the following were present: (1) refractory shock (i.e., patients expected to die within the first 24 h), (2) an inability to achieve source control (i.e., irreversible disease states such as unresectable dead bowel), (3) pre-sepsis expected lifespan < 3 months, (4) patient/family not committed to aggressive management, (5) severe CHF (NYHA class IV), (6) Child-Pugh class C liver disease or pre-liver transplant, (7) known HIV with CD4^+^ count < 200 cells/mm^3^, (8) organ transplant recipient or use of chronic corticosteroids or immunosuppressive agents, (9) pregnancy, (10) institutionalized patients, (11) chemotherapy or radiotherapy within 30 days, (12) severe traumatic brain injury (i.e., evidence of neurological injury on CT scan and a GCS < 8), (13) spinal cord injury resulting in permanent sensory and/or motor deficits, or (14) inability to obtain informed consent.

### Patient classification

Patients were diagnosed with sepsis, severe sepsis, or septic shock using the definitions established by the Society of Critical Care Medicine, the European Society of Intensive Care Medicine, the American College of Chest Physicians, the American Thoracic Society, and the Surgical Infection Society (SCCM/ESICM/ACCP/ATS/SIS) 2001 International Sepsis Definitions Conference [39]. CCI was defined as an ICU length of stay (LOS) greater than or equal to 14 days with evidence of persistent organ dysfunction, measured using components of the Sequential Organ Failure Assessment (SOFA) score (i.e., cardiovascular SOFA ≥ 1 or score in any other organ system ≥ 2) [40]. Patients with an ICU LOS less than 14 days would also qualify for CCI if they were discharged to another hospital, to a long-term acute care facility, or to a hospice and demonstrated continuing evidence of organ dysfunction at the time of discharge. Those patients experiencing death within 14 days of sepsis onset were excluded from the clinical and biomarker analyses. Any patient who did not meet the criteria for CCI or early death was classified as rapid recovery. Since there is no consensus definition for CCI, we focused on combining key elements established by previous definitions reported in the literature, including the requirement for prolonged intensive care and the presence of persistent organ dysfunction. However, our definition was modified to include a broader classification of organ dysfunction, as previous definitions relied heavily on the presence of respiratory failure requiring mechanical ventilation.

### Human blood collection

EDTA-anticoagulated human whole blood samples were collected by venipuncture on days 1 (*n* = 241), 4 (*n* = 211), 7 (*n* = 166), 14 (*n* = 100), 21 (*n* = 59), 28 (*n* = 38), 35 (*n* = 28), and 42 (*n* = 18) meeting the Sepsis-2 criteria. Of these, 23 subjects were used for MDSC and T lymphocyte co-culture experiments, while 117 septic and 11 control subject samples were utilized for miR isolation and analysis. This discrepancy in the sample number is a result of the different blood volumes and availability required for the different analyses. Samples were stored on ice and processed within 6 h after blood drawing. An additional cohort of de-identified patient blood samples [20] from previously published data, stored in − 80 °C freezer, was analyzed for MDSC genome-wide expression analysis. This study included 74 patients meeting the Sepsis-2 severe sepsis or septic shock criteria who were sampled at identical time points, as well as from 18 control subjects [24, 36].

### MDSC sorting and phenotypic analysis

**Fig. 1:**
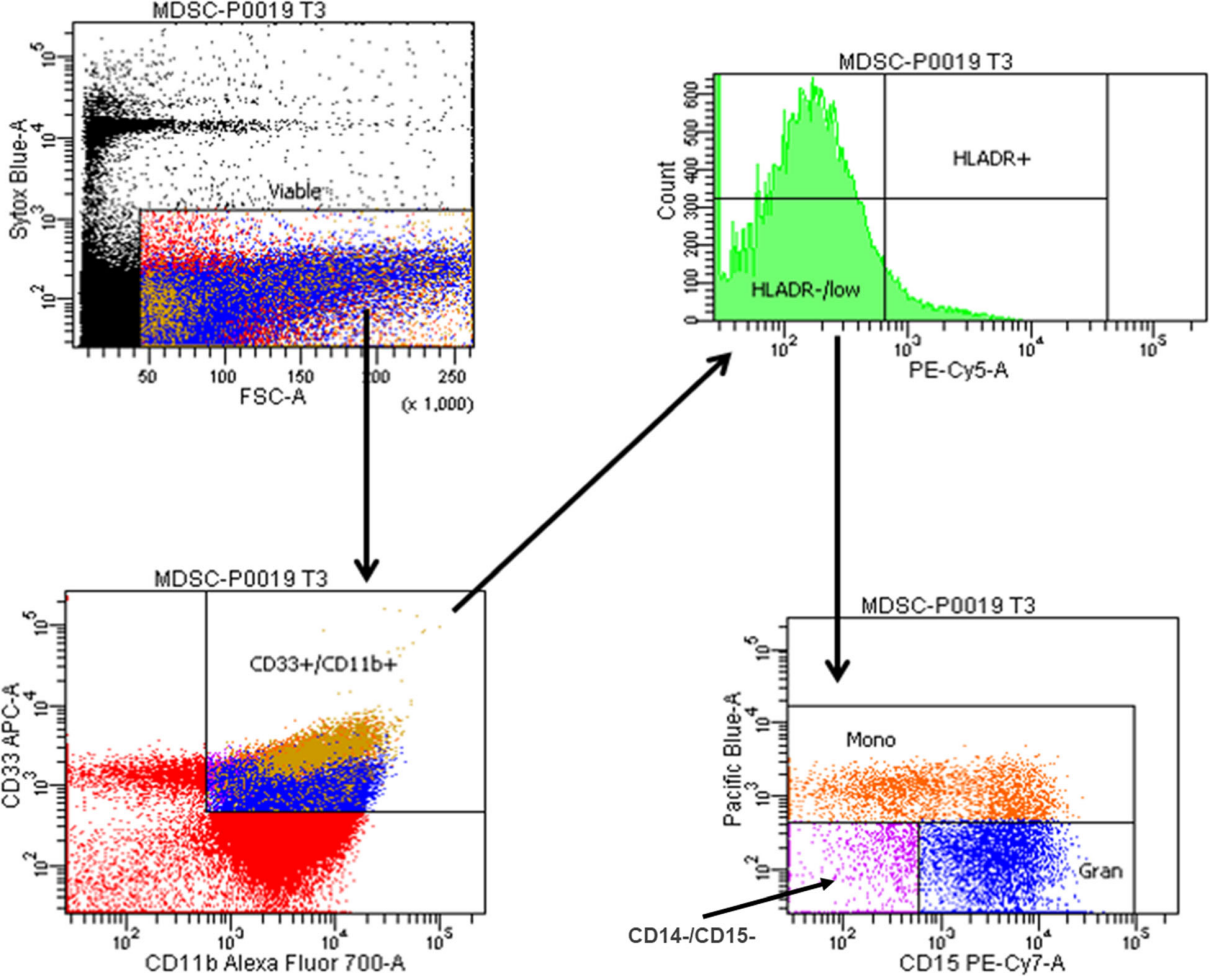
Phenotypic classification of MDSCs. Gating strategy used to classify CD33^+^CD11b^+^HLA-DR^−/low^ MDSC populations in the human whole blood using samples stained according to the protocol and analyzed on the LSR II flow cytometer. Monocytic MDSC subpopulations were further characterized as CD14^+^ and granulocytic MDSCs as CD14^−^CD15^+^

Human whole blood samples were labeled with CD33^+^ conjugated to APC, CD11b^+^ conjugated to Alexa Fluor 700, HLA-DR conjugated to PE-Cy5, CD14^+^ conjugated to Pac Blue, and CD15^+^ conjugated to PE-Cy7. The samples were analyzed on the LSR II flow cytometer (Becton-Dickinson), and MDSCs were characterized as CD33^+^CD11b^+^HLA-DR^low/−^ (Fig. 1). Monocytic MDSCs (M-MDSCs) were further characterized as CD14^+^ and granulocytic MDSCs (G-MDSCs) as CD14^−^CD15^+^ (Fig. 1), while non-monocytic, non-granulocytic MDSCs were CD14^−^CD15^−^. Absolute counts of MDSCs were calculated using total white blood cell count (cells/μl) and the fraction of MDSCs from total viable cells. The normal range of MDSC absolute count from healthy control subjects was calculated using mean values of reported total leukocyte counts.

Whole blood samples from septic patients on days 4 (*n* = 7), 7 (*n* = 5), 14 (*n* = 7), and 21 (*n* = 4) were stained using the same staining protocol above, and CD33^+^CD11b^+^HLA-DR^low/−^ cells were sorted on the Bio-Rad S3e cell sorter. Cell sort purity (≥ 90%) was verified on the LSR II flow cytometer. Sorted MDSCs from septic patients were utilized for functional T cell proliferation/suppression culture assays as well miR analysis.

### Human T cell isolation and proliferation assay

Peripheral blood mononuclear cells (PBMC) from the human whole blood from septic patients were collected simultaneously using Histopaque™ (Sigma-Aldrich, St. Louis, MO) and density gradient centrifugation. Total T cells in the suspension were captured by immunomagnetic negative selection using EasySep™ Human T Cell Isolation Kit (Stemcell Technologies, Vancouver) according to the manufacturer’s instructions. Isolated CD3^+^ T lymphocytes were labeled with cell trace violet (Thermo Fisher, Waltham, MA) to detect for T cell proliferation.

T lymphocytes (1 × 10^5^ CD3^+^) were seeded into a 96-well plate and stimulated with 25 μl/ml soluble anti CD3/CD28 antibodies (concentration of the activator is proprietary information; Stemcell Technologies, Vancouver), according to the manufacturer’s protocol [41]. Prior to these experiments, we conducted a titration curve. We conducted three serial dilutions of CD3/28 concentrations in order to determine proper stimulation dosage (Additional file 1: Figure S1). Isolated T lymphocytes were plated and stimulated with 1:4, 1:2, and undiluted concentrations of the soluble CD3/28 preparation and cultured for 4 days at 37 °C and 5% CO_2_ per recommendation of the manufacturer. Unstimulated T cells served as control for these experiments.

Once an optimal CD3/CD28 concentration was determined for ex vivo T lymphocyte stimulation, an aliquot of these cells was co-cultured with MDSCs in a 1:1 ratio at 37 °C and 0 5% CO_2_. After 4 days, the cells were harvested and the supernatant was obtained for cytokine analysis. Cells were stained with anti-CD8 conjugated to FITC and anti-CD4 conjugated to PE. Fluorescence in the cell population was detected by flow cytometry (LSR II, Becton Dickinson, Franklin Lakes, NJ) as previously described [20].

A proliferation index was calculated as the total number of divisions divided by the number of cells that went into division [42]. The proliferation index takes into account only the cells that underwent at least one division, i.e., only responding cells are reflected in the proliferation index (http://docs.flowjo.com/vx/experiment-based-platforms/proliferation/plat-prolif-protocols/). The percent suppression by MDSCs was calculated using the following formula: [100 − (proliferation index stimulated T cells + MDSC/proliferation index stimulated T cells) 100)].

### Cytokine analysis

Human high sensitivity T cell magnetic bead 6-plex panel (IFNγ, IL-10, IL-12 (p70), IL-17α, IL-2, IL-23) was purchased from EMD Millipore (Billerica, MA). Supernatants after cell culture were used for T cell-associated cytokines in septic patients. The xPONENT software (EMD Millipore, Billerica, MA) was used for cytokine analysis.

### Transcriptomic profile analysis

MDSCs were isolated from the whole blood as described above for functional assays. RNA was extracted from lysates using QIAGEN Rneasy™ Mini Kit (Qiagen), labeled and hybridized onto GeneChip® Human Transcriptome Array 2.0 (Affymetrix, Santa Clara, CA), and processed following the manufacturer’s instructions. BRBArray Tools® was used to identify significant microarray gene expression differences. Fold expression changes of the significant genes were calculated over age/sex-matched controls. The significant genes were further analyzed with Ingenuity Pathway Analysis (IPA) software™. IPA software was employed to make downstream functional predictions from these groups of genes with a *Z*-score greater than two indicating significance.

### miR expression and predicted miR target gene prediction

miR expression from enriched MDSCs was analyzed using the TaqMan Advanced miR cDNA Synthesis Kit (Thermo Fisher Scientific, Carlsbad, CA) (*n* = 94). miR expression patterns were calculated with a log2-transformed expression matrix with significant expression differences (fold expression changes over age/sex-matched control) identified using BRBArrayTools® (*p* < 0.05). Predicted target genes of the differentially expressed miR were identified with TargetScan Human 7.2 [43] which predicts biological targets of miRs, by searching for conserved 8mer, 7mer, and 6mer sites matching the seed region of each miR.

### Statistical analysis

Results for continuous variables are reported as mean ± SD for normally distributed variables or median ± inter-quartile range (IQR) for non-normally distributed variables. Normality was checked via the Shapiro-Wilk test. Student’s *t* test or non-parametric Mann-Whitney test was used to compare normal or non-normal variables, respectively, between different groups or time points. Tukey’s multiple comparison procedure was used to adjust *p* values for multiple comparisons. Data were analyzed using Prism 7 (GraphPad Software, CA) and SAS 9.4 (SAS Institute Inc., Cary, NC).

## Results

Among the 262 sepsis patients included in flow cytometric analysis, the mean age was 59 years, and 121 (46%) were female. Two hundred forty-six (94%) of these patients had at least 1 comorbid condition, and the median number of comorbidities was 2.

### MDSCs are present and persistent in surgical sepsis patients

**Fig. 2:**
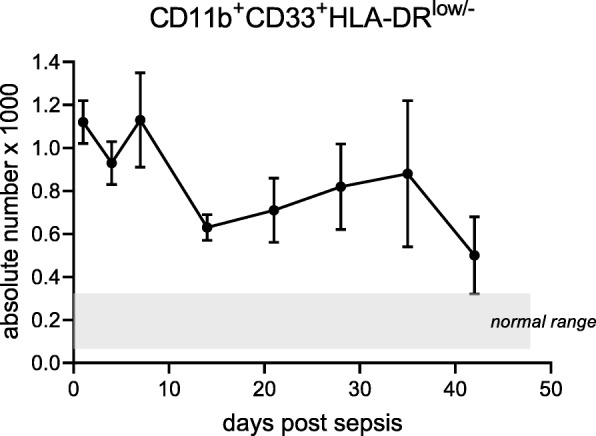
Absolute counts of circulating myeloid-derived suppressors in acute and chronic sepsis. Cells with the surface markers CD33^+^CD11b^+^HLA-DR^−/low^ were identified on the flow cytometer, and absolute counts ([total white blood cell count (cells/ml) × percent CD33^+^CD11b^+^HLA-DR^low/−^]/100) were determined for septic patients. The mean values of absolute counts are reported for patients at various time points beginning 24 h (*n* = 241) after the initial infectious onset and on days 4 (*n* = 211), 7 (*n* = 166), 14 (*n* = 100), 21 (*n* = 59), 28 (*n* = 38), 36 (*n* = 28), and 42 (*n* = 18) in patients meeting the Sepsis-3 criteria

Similar to our and others’ previous results [20, 44], immature myeloid cells in the blood were both present and persistent following sepsis. These cells remained noticeably elevated above the numbers of immature myeloid cells in healthy controls for up to 42 days following the septic infectious insult (Fig. 2). Interestingly, the percentage of circulating MDSCs (defined by cell surface phenotype, i.e., CD33^+^CD11b^+^HLA-DR^low/−^) in septic patients was consistent with the range demonstrated to be present in the circulation of cancer patients [45, 46].

Flow cytometric analysis revealed that septic patients (*n* = 211) 4 days following sepsis had a median percentage (relative population) of 22.4% CD33^+^CD11b^+^HLA-DR^low/−^CD14^+^ M-MDSCs and 7.9% CD33^+^CD11b^+^HLA-DR^low/−^CD14^−^ G-MDSCs (Table 1). The remaining 69.7% of cells were CD33^+^CD11b^+^HLA-DR^low/−^CD14^−^CD15^−^ (Table 1). These cells are potentially the “early MDSCs (E-MDSCs)” described by Veglia et al. [47]. In addition, these cells are potentially immature myeloid cells with the potential to differentiate into other MDSC populations or fully differentiated innate immune cells [48]. Finally, there is a subset of G-MDSCs that can be CD14^−^CD15^−^ [47]. Although not significant, 14 days after sepsis, survivors still hospitalized (*n* = 100) trended toward a decreased percentage of M-MDSCs (17.8%) vs control (21.5%). There was a near doubling in the number of circulating G-MDSCs (5.6 to10.6%) 14 days after sepsis as compared to control subjects. Additionally, the percentage of M-MDSCs and G-MDSCs present at day 14 vs day 4 decreased and increased, respectively (*p* < 0.05, Table 1).

**Table 1:** Phenotypic analysis of MDSCs from septic and control patients

	% MDSC (of total leukocytes)	% M-MDSC (of total MDSCs)	% G-MDSC (of total MDSCs)	% CD14^−^CD15^−^MDSC (of total MDSCs)
Control (Q1, Q3)	3.00 (0.6, 5.0)	21.50 (8.7, 55.5)	5.60 (3.0, 12.2)	72.90
Sepsis day 4 (Q1, Q3)	4.50* (2.2, 8)	22.40 (12.5, 41.2)	7.90 (1.5, 19.3)	69.70
Sepsis day 14 (Q1, Q3)	4.90* (1.95, 8.55)	17.85^*#*^ *(10.2, 30.4)*	10.55*^,#^ *(3.4, 30.60)*	71.60

*% MDSC* all myeloid-derived suppressor cell, *M-MDSC* monocytic myeloid-derived suppressor cell, *G-MDSC* granulocytic myeloid-derived suppressor cell

*p < 0.05 vs control

^#^*p* < 0.05 vs day 4

### MDSC function evolves over time after sepsis

Subset phenotypic analysis of MDSCs specifically isolated and utilized for the suppression assays revealed a decrease in the median value of absolute MDSC counts from patient samples obtained at day 4 (258/mm^3^) to samples from day 14 (219 /mm^3^). The median percentage (Q1, Q3) of MDSCs found in the blood of these patients increased slightly from 2.7% (1.8, 5.8) at day 4 to 4.3% (2.4, 4.6) at day 14. Patients at day 4 following sepsis (*n* = 7) had a median value of 34.0% M-MDSCs, which decreased to 4.3% by day 14, but the difference was not significant. Over time, the median percentage of granulocytic MDSCs increased, but again was not significant, specifically being 10.9% G-MDSCs at day 4 and 26.2% G-MDSCs at day 14.

**Fig. 3:**
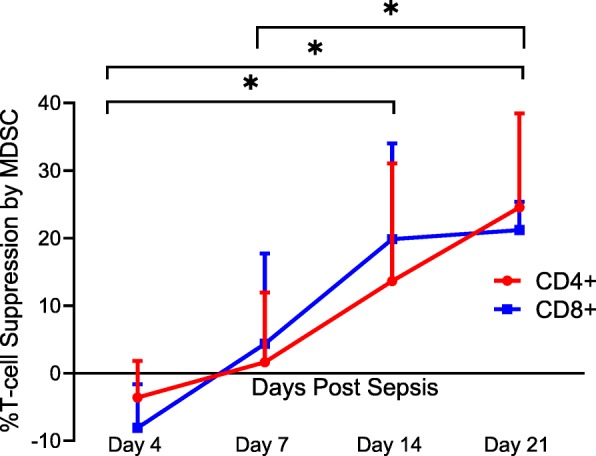
Percent T lymphocyte suppression by MDSCs. Immature myeloid cells with the surface markers CD33^+^CD11b^+^HLA-DR^−/low^ were isolated on days 4, 7, 14, and 21 after sepsis. Autologous T lymphocytes were stimulated with soluble anti-CD3/28 and seeded in a co-culture with MDSCs in a 1:1 ratio. T cells were labeled with CellTrace Violet to detect for proliferation, and a proliferation index (PI) was calculated for both CD4^+^ and CD8^+^ T cells using flow cytometric analysis. Percent suppression was calculated as the ratio of PIs from stimulated T cells in the presence of MDSCs and the PI of stimulated T cells in culture medium alone. Percent suppression for both CD4^+^ and CD8^+^ T cells was significantly different between day 4 vs 14 (*p* = 0.0402 and 0.0012), day 4 vs 21 (*p* = 0.0225 and < 0.0001), and day 7 vs 21 (*p* = 0.037 and 0.045). There was no significance noted of percent suppression of CD4^+^ and CD8^+^ T cells between days 7 and 14 (*p* = 0.17 and 0.08)

Immature myeloid cells that met the cell surface definition of human MDSCs [49] did not have a similar capacity to suppress T lymphocyte proliferation at different time points after sepsis. Much to our surprise, CD33^+^CD11b^+^HLA-DR^low/−^ cells isolated on day 4 after sepsis displayed a stimulatory, rather than suppressive, effect on lymphocyte proliferation to CD3/CD28 stimulation (Fig. 3). Only at or after 14 days following sepsis onset did the isolated CD33^+^CD11b^+^HLA-DR^low/−^ cells display the T cell-suppressive activity required to be classified as MDSCs (Fig. 3).

**Fig. 4:**
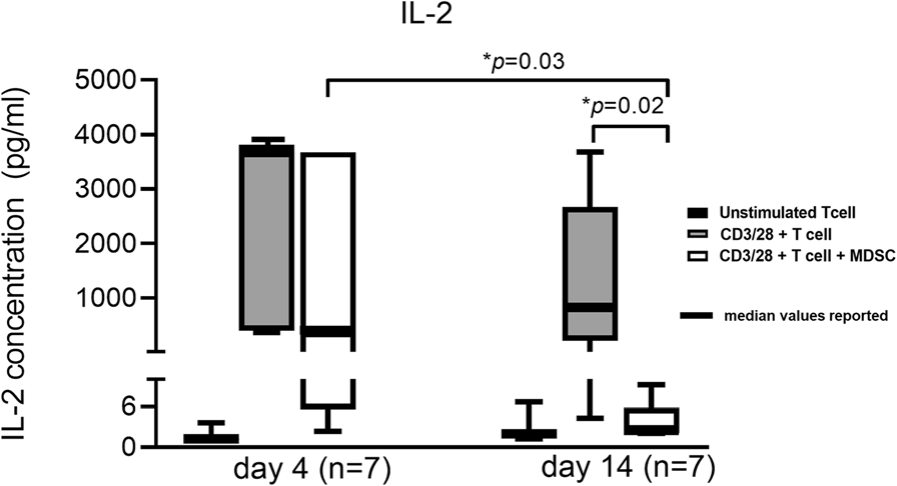
IL-2 concentration in functional T cell proliferation and suppression assay supernatants. After 4 days of cell culture in the following groups: (1) unstimulated T cells in culture medium, (2) CD3/28-stimulated T cells in culture medium, and (3) CD3/28-stimulated T cells in the presence of culture medium and MDSCs (1:1), supernatant was collected for quantification of IL-2 concentration (pg/ml) in septic patients from days 4 (*n* = 7) and 14 (*n* = 7). Concentration levels of IL-2 were significantly different (*p* = 0.03) between stimulated T cells at days 4 and 14 and between stimulated T cells and stimulated T cells + MDSCs at day 14 (*p* = 0.02)

**Fig. 5:**
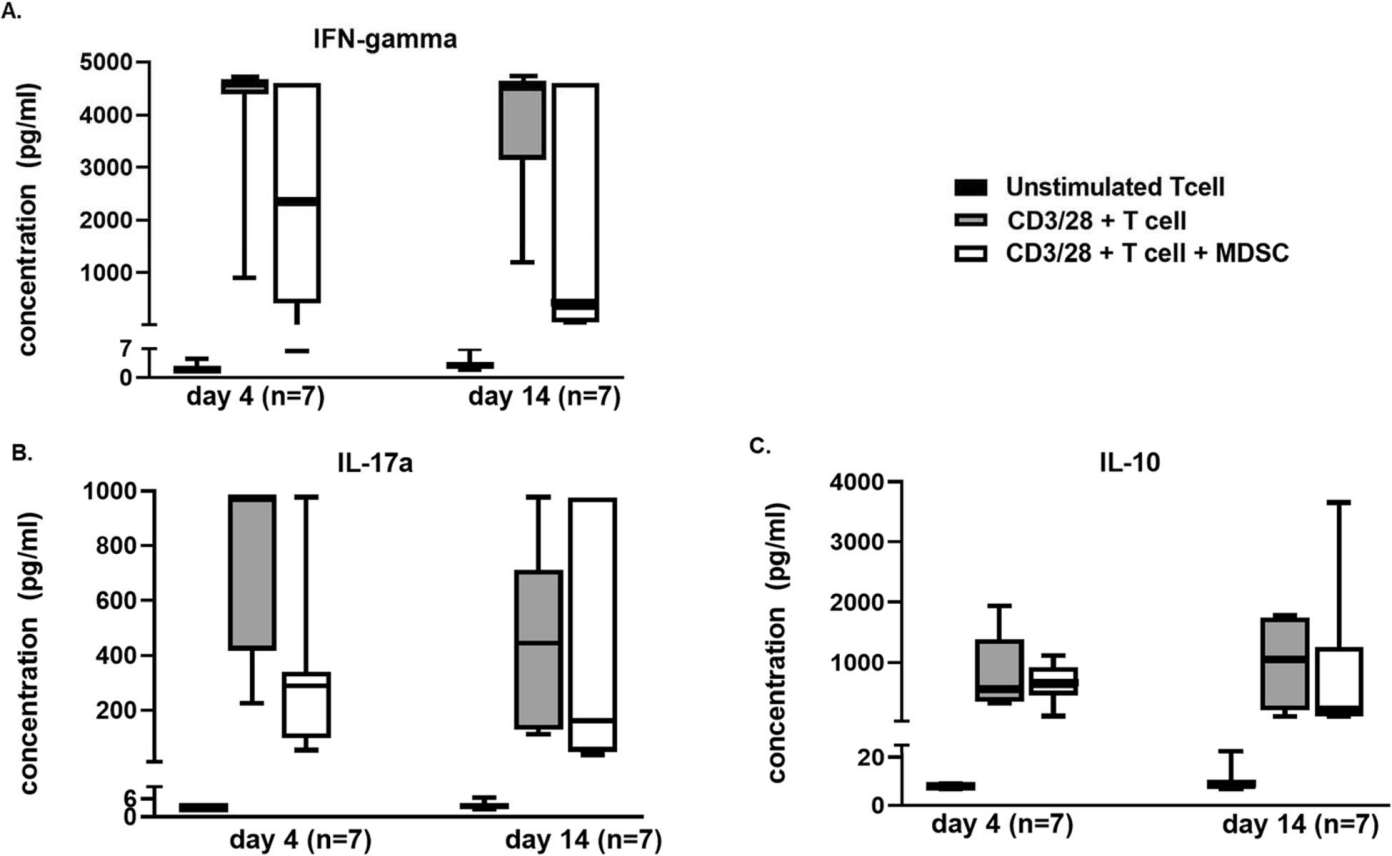
Cytokine concentrations in functional T cell proliferation and suppression assays. After 4 days of cell culture in the following groups: (1) unstimulated T cells in culture medium, (2) CD3/28-stimulated T cells in culture medium, and (3) CD3/28-stimulated T cells in the presence of culture medium and MDSCs (1:1), supernatant was collected for quantification of interferon-γ (**a**), interleukin-17α (**b**), and interleukin-10 (**c**) concentration (pg/ml) in septic patients from days 4 (*n* = 7) and 14 (*n* = 7). No significant differences were found

Importantly, MDSCs also altered the production of T lymphocyte-specific cytokines in response to CD3/CD28 stimulation. Co-culturing day 14 MDSCs with T lymphocytes significantly suppressed interleukin-2 (IL-2) concentrations in the supernatant after 4 days (*p* = 0.02) (Fig. 4). However, co-culture with day 4 isolated MDSCs did not have a significant effect on total IL-2 production (*p* = 0.15). There was no difference in the other cytokines (IFN-γ, IL-10, and IL-17α) measured after MDSC:T lymphocyte co-culture with CD3/CD28, although there was a trend toward suppressed IFN-γ concentrations after 14 days (Fig. 5a–c). Though IL-10 is an important product of T_H2_ cells, it is also a known product of MDSCs; therefore, it is not surprising that IL-10 levels were not significantly depleted in the presence of MDSCs [50].

### MDSC miRNA expression patterns alter over time after sepsis

A comparison of MDSC miRNA from septic (*n* = 61) and control (*n* = 11) subjects revealed 216 miRNAs that were differentially expressed (significant fold changes as compared to control subject expression) at all the time points (i.e., days 4, 14, 21, 28, 35, and 42). At day 4 post-sepsis, the expression of 58 unique miRNAs displayed significant downregulation when compared to control subjects, while the expression of the remaining 158 were significantly upregulated. Forty-two distinct miRNAs from day 4 patients demonstrated at least a twofold positive or negative change in the expression when compared with control subjects (Table 2). At day 14 post-sepsis, the expression of 82 miRNAs were significantly downregulated and 134 were significantly upregulated. Of these, 27 miRNAs expressed significant fold changes ≥ 2 (Table 3). Table 4 displays the 20 miRNAs with the greatest absolute values of fold change across both time points day 4 and 14 post-sepsis. Importantly, 48 of the same unique miRNAs had significantly different expression at days 4 and 14 following sepsis, which were the days that functional differences in the post-septic circulating MDSCs were identified. Among these were miRNAs 17, 21, 106, and 181, all of which are known to influence MDSC function and persistence (Table 5).

**Table 2:** Significant fold changes │≥2│ in miRNA expression patterns 4 days following sepsis

miRNA	Fold change
Upregulated	
hsa-miR-27a-3p	3.7
hsa-miR-3940-5p	2.9
hsa-miR-30c-5p	2.6
hsa-miR-6821-5p	2.5
hsa-miR-5100	2.5
hsa-miR-145-5p	2.5
hsa-let-7f-5p	2.5
hsa-miR-4739	2.4
hsa-miR-222-3p	2.4
hsa-miR-6805-5p	2.3
hsa-miR-15a-5p	2.3
hsa-miR-8069	2.2
hsa-miR-7108-5p	2.2
hsa-miR-6752-5p	2.2
hsa-miR-4516	2.2
hsa-miR-223-3p	2.2
hsa-miR-221-3p	2.2
hsa-miR-6816-5p	2.1
hsa-miR-6791-5p	2.1
hsa-miR-574-3p	2.1
hsa-miR-4707-5p	2.1
hsa-miR-425-5p	2.1
hsa-miR-18a-5p	2.1
hsa-let-7 g-5p	2.1
hsa-miR-4467	2.0
hsa-miR-4466	2.0
hsa-miR-4281	2.0
hsa-miR-3621	2.0
hsa-miR-3178	2.0
hsa-miR-143-3p	2.0
Downregulated	
hsa-miR-6868-5p	− 2.0
hsa-miR-4530	− 2.0
hsa-miR-5189-3p	− 2.0
hsa-miR-3921	− 2.1
hsa-miR-4791	− 2.1
hsa-miR-548ap-3p	− 2.3
hsa-miR-4668-5p	− 2.4
hsa-miR-3613-3p	− 2.4
hsa-miR-3942-5p	− 3.2
hsa-miR-1263	− 3.6
hsa-miR-200b-5p	− 3.8
hsa-miR-1298-3p	− 4.3

**Table 3:** Significant fold changes │≥2│ in miRNA expression patterns 14 days following sepsis

miRNA	Fold change
Upregulated	
hsa-miR-6821-5p	2.8
hsa-miR-6085	2.6
hsa-miR-5000-5p	2.5
hsa-miR-4707-5p	2.5
hsa-miR-8069	2.2
hsa-miR-27a-3p	2.2
hsa-miR-223-3p	2.2
hsa-miR-3940-5p	2.1
hsa-miR-7108-5p	2
hsa-miR-6800-5p	2
hsa-miR-6791-5p	2
hsa-miR-6752-5p	2
hsa-miR-30c-5p	2
Downregulated	
hsa-miR-1246	− 2.1
hsa-miR-3175	− 2.1
hsa-miR-365b-5p	− 2.1
hsa-miR-4443	− 2.1
hsa-miR-3921	− 2.2
hsa-miR-4708-3p	− 2.2
hsa-miR-4791	− 2.2
hsa-miR-6868-5p	− 2.6
hsa-miR-548ap-3p	− 2.9
hsa-miR-1263	− 3
hsa-miR-3942-5p	− 4.1
hsa-miR-7641	− 4.4
hsa-miR-1298-3p	− 5
hsa-miR-200b-5p	− 5.1

**Table 4:** Top up- and downregulated miRNA expression in septic human MDSCs at days 4 and 14

	Fold change
Day 4	
Upregulated	
hsa-miR-27a-3p	3.7
hsa-miR-3940-5p	2.9
hsa-miR-30c-5p	2.6
hsa-miR-5100	2.5
hsa-miR-145-5p	2.5
hsa-let-7f-5p	2.5
hsa-miR-6821-5p	2.5
hsa-miR-222-3p	2.4
hsa-miR-4739	2.4
hsa-miR-6805-5p	2.3
Downregulated	
hsa-miR-6868-5p	− 2.0
hsa-miR-3921	− 2.1
hsa-miR-4791	− 2.1
hsa-miR-548ap-3p	− 2.3
hsa-miR-3613-3p	− 2.4
hsa-miR-4668-5p	− 2.4
hsa-miR-3942-5p	− 3.2
hsa-miR-1263	− 3.6
hsa-miR-200b-5p	− 3.8
hsa-miR-1298-3p	− 4.3
Day 14	
Upregulated	
hsa-miR-6821-5p	2.8
hsa-miR-6085	2.6
hsa-miR-5000-5p	2.5
hsa-miR-4707-5p	2.5
hsa-miR-8069	2.2
hsa-miR-27a-3p	2.2
hsa-miR-223-3p	2.2
hsa-miR-3940-5p	2.1
hsa-miR-7108-5p	2.0
hsa-miR-30c-5p	2.0
Downregulated	
hsa-miR-4791	− 2.2
hsa-miR-4708-3p	− 2.2
hsa-miR-3921	− 2.2
hsa-miR-6868-5p	− 2.6
hsa-miR-548ap-3p	− 2.9
hsa-miR-1263	− 3.0
hsa-miR-3942-5p	− 4.1
hsa-miR-7641	− 4.4
hsa-miR-1298-3p	− 5.0
hsa-miR-200b-5p	− 5.1

**Table 5:** Myeloid cell effect of specific MDSC miRNAs

miRNA	Day 4	Day 14	Function
hsa-miR-21-5p	↑	↓	Expressed in myeloid cells. Elevated levels enhance proliferation and block myeloid differentiation (Zhang et al. 2014. *JI*)
hsa-miR-17-5p	↑	↓	Plays an important role in regulating the suppressive potential of MDSCs via STAT3 (Zhang et al. 2011. *JI*)
hsa-miR-181a-5p	↑	↓	Expressed in myeloid cells. Elevated levels are known to induce MDSC proliferation (McClure et al. 2014. *ASM*)
hsa-miR-106a-5p	↑	↓	Influences myeloid development. Overexpression represses M-CSFR and limits myeloid differentiation (El-Gazzar et al. 2013. *Innate Immunity*)

The myeloid cell effects of the specific MDSC miRNAs that were found to have different expression patterns in MDSCs from septic patients at says 4 and 14 after initial infection

### miRNAs are differentially expressed according to sepsis severity (sepsis vs septic shock) or septic patient outcome (CCI vs RAP)

**Fig. 6:**
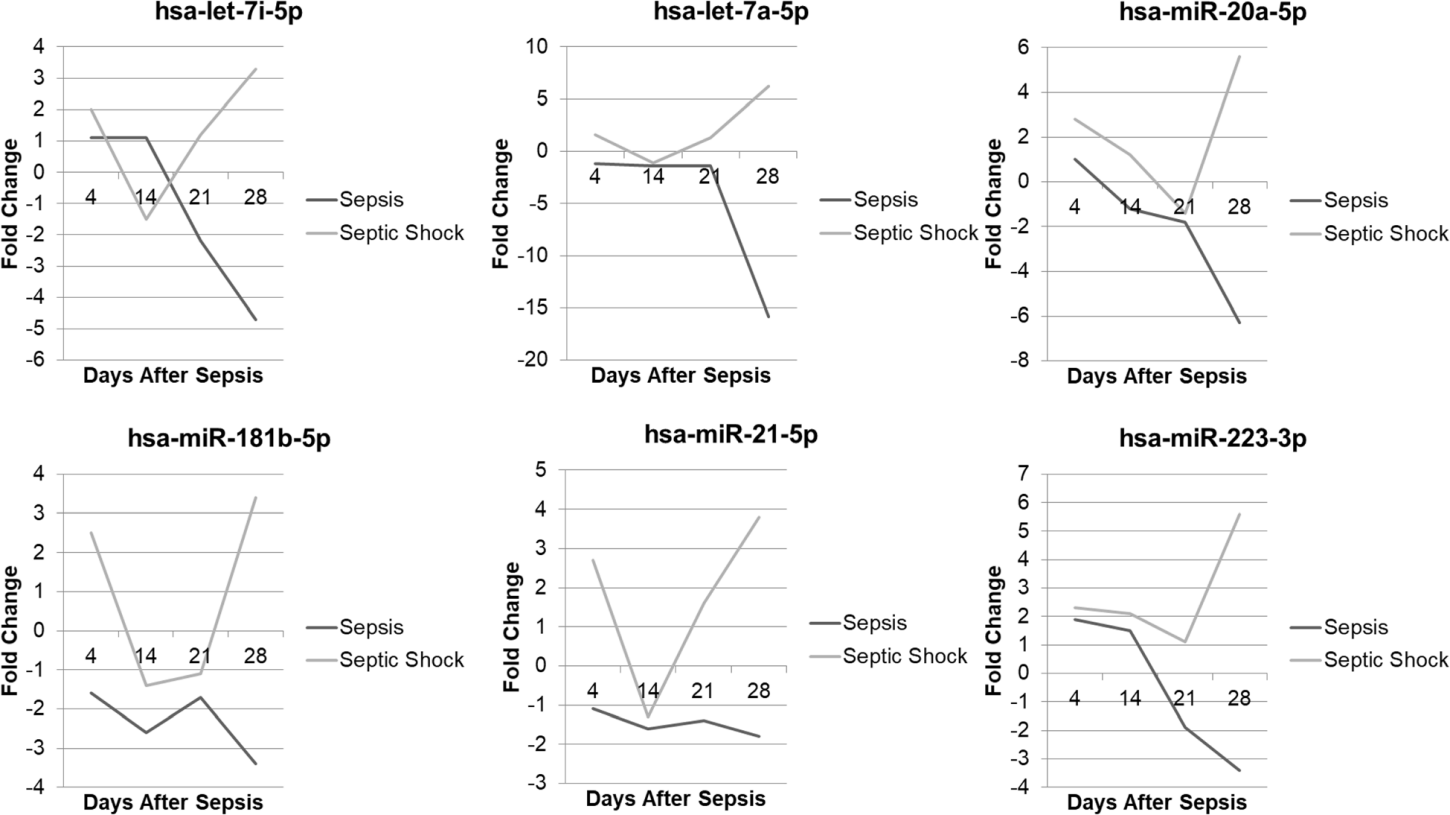
A comparison of miRNA expression patterns between patients with septic shock vs sepsis. MDSCs from patients with septic shock had significantly different miRNA expression patterns (fold change difference ≥ 2) as compared to patients with sepsis at or beyond day 14 following sepsis

Sepsis is defined as life-threatening organ dysfunction caused by a dysregulated host response to infection. Septic shock accounts for a subset of these patients with persistent hypotension-need for vasopressors to maintain a mean arterial blood pressure ≥ 65 mmHg and a lactate level > 2 mmol/l, despite adequate fluid resuscitation [2]. Importantly, septic shock, a known risk factor for worse patient outcomes [51], induced differential expression of 194 significant miRs in MDSCs from subjects with sepsis (without shock). Among these were the miRs let-7, 20b, 21, 181b, and 223, all associated with MDSC function and persistence. At 21 days after septic shock, these miRNAs were still upregulated in MDSCs as compared to MDSCs isolated at 21 days after sepsis (without shock) (Fig. 6).

**Fig. 7:**
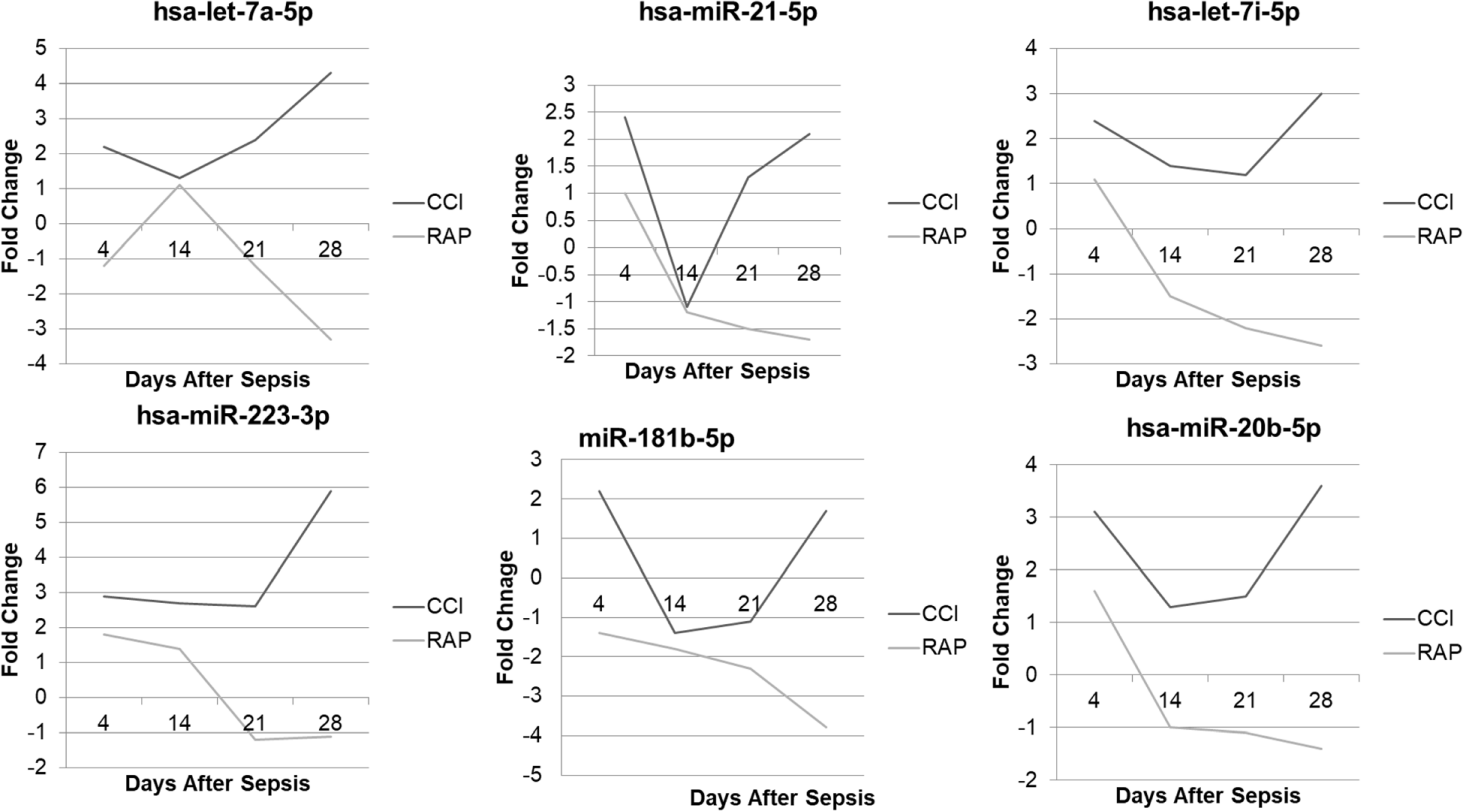
A comparison of miR expression patterns between patients with CCI vs RAP after surgical sepsis. MDSCs from patients with CCI had significantly different miR expression patterns (fold change difference ≥ 2) as compared to patients who rapidly recovered from sepsis at or beyond day 14 following sepsis

Similarly, septic patients who developed CCI displayed significant fold changes in microRNAs that were different when compared to those from patients who rapidly recovered (RAP) following sepsis. We identified 215 miRNAs that were differentially expressed across every time point in MDSCs from 4 to 42 days after sepsis. Interestingly, many of the miRNAs with the greatest fold changes between CCI and RAP patients on day 14 are known to be important to the MDSC function (Table 6). Additionally, all of these microRNAs were upregulated in CCI and downregulated in RAP at day 14. Of these microRNAs, several are known to influence myeloid cells in the murine and cancer literature, and, similar to septic shock, some of these were found to be differentially expressed in the MDSCs isolated from patients who experienced CCI vs rapid recovery (Fig. 7). The similarity of CCI to septic shock is not surprising as septic shock is an independent risk factor for the development of CCI [6].

**Table 6:** miR in human MDSCs from CCI vs RAP patients at day 14 with largest absolute fold change (FC) difference

Day 14	CCI fold change	RAP fold change	Absolute FC difference
hsa-miR-17-5p	1.1	− 2.6	3.7
hsa-miR-181a-5p	1	− 2.6	3.6
hsa-miR-23b-3p	2.3	− 1.1	3.4
hsa-miR-103a-3p	1	− 2.1	3.1
hsa-miR-4270	2	− 1.1	3.1
hsa-miR-106a-5p	1.2	− 1.9	3.1
hsa-miR-1343-5p	1.7	− 1.3	3
hsa-miR-191-5p	1.4	− 1.6	3
hsa-miR-25-3p	1.7	− 1.3	3
hsa-miR-3124-5p	1.3	− 1.6	2.9
hsa-miR-6126	1.3	− 1.6	2.9
hsa-miR-15b-5p	1.8	− 1.1	2.9
hsa-miR-4651	1.7	− 1.2	2.9
hsa-miR-3178	1.7	− 1.2	2.9
hsa-let-7i-5p	1.4	− 1.5	2.9
hsa-miR-106b-5p	1.5	− 1.4	2.9
hsa-miR-425-5p	1.9	− 1	2.9
hsa-miR-4758-5p	1.7	− 1.1	2.8
hsa-miR-6798-5p	1.6	− 1.2	2.8
hsa-miR-7977	1.3	− 1.5	2.8

### Genome-wide expression differences are present in MDSCs from severe sepsis/septic shock patients at day 14 following infectious onset

**Fig. 8:**
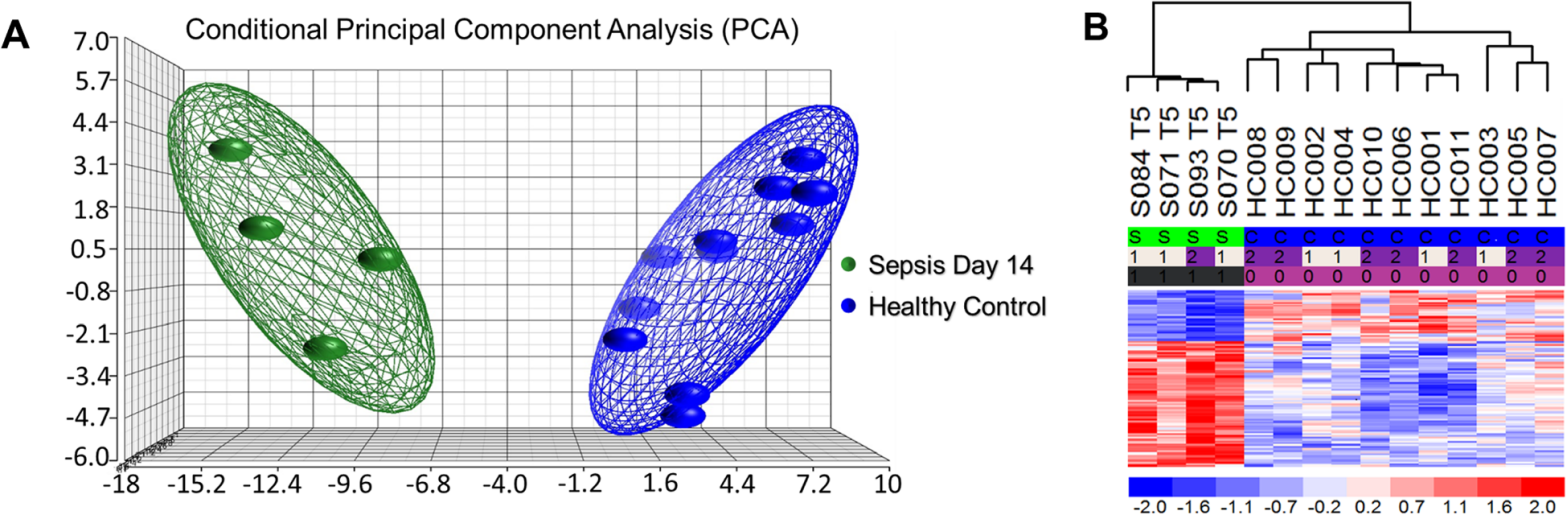
Microarray transcriptomic analysis of MDSCs from patients 14 days after sepsis and healthy control subjects. The genomic response of isolated MDSC RNA in healthy controls and septic patients 14 days after initial infectious onset. **a** Conditional principal component analysis of septic (day 14) and healthy control myeloid-derived suppressor cell gene expression patterns. **b** Heat map of the hierarchical clustering of MDSC gene expression patterns and variation between septic patients from day 14 and healthy control subjects

Transcriptional analysis of a separate patient cohort [20] at day 14 revealed significant genomic differences from circulating MDSCs (Fig. 8a, b). A total of 95 genes were found to be differentially expressed in MDSCs from septic patients at day 14 when compared to CD33^+^CD11b^+^HLA-DR^−/low^ cells isolated from healthy controls. Of these, 68 genes were found to be significantly overexpressed, while the remaining 27 displayed significant downregulation (Table 7). Among those discovered were the following genes associated with MDSC proliferation and MDSC immunosuppressive function: *ARG1* (↑), *CEACAM* (↑), *FKBP5* (↑), *HMGB2* (↑), *LCN2* (↑), *MMP8/9* (↑), *MS4A4A* (↑), *PFKFB3* (↑), *RETN* (↑), *S100A8* (↑), *VCAN-AS1* (↑), *CLL4* (↓), *HLA-DQB1* (↓), *HLA-DRB1* (↓), and *IL7R* (↓) [44, 52, 53]*.*

**Table 7:** Transcriptomic differences in MDSCS isolated 14 days after severe sepsis/septic shock compared to healthy controls

Expression	Gene name
Upregulated	*ABCA13*, *ACVR1B*, *ADAM9*, *ANKRD34B*, *ANXA3*, ***ARG1***, *ARID5B*, *C19orf59*, *CD177*, *CD63*, *CDADC1*, ***CEACAM1***, *CLU*, *DACH1*, *ELMO2*, *FAM20A*, *FBXO9*, *FBXW2*, *FCAR*, ***FKBP5***, *GADD45A*, *GCLM*, *GCNT1*, *GPR141*, *GPR84*, *GSR*, *GYG1*, *HK3*, ***HMGB2***, *HP*, *HPR*, *KIF1B*, ***LCN2***, *LILRA5*, *LTF*, *MACF1*, *MAP 2K6*, *MAPK14*, *METTL7B*, *MIR4441*, *MIR4677*, *MKI67*, ***MMP8***, ***MMP9***, ***MS4A4A***, *MS4A4E*, *PAG1*, *PECR*, *PFKFB2*, ***PFKFB3***, *PLAC8*, ***RETN***, *RGL4*, *RN5S22*, ***S100A8***, *SAMSN1*, *SEL1L3*, *SLC26A8*, *SLC36A1*, *SNORA79*, *SYNE1*, *TCN1*, *TDRD9*, *TMEM14E*, *UGCG*, *UPP1*, ***VCAN-AS1***, *ZNF407*
Downregulated	*CACNA2D3*, ***CCL4***, *CDKN1C*, *CHST7*, *ENOSF1*, *FAIM3*, *FCER1A*, *GZMA*, *HES1*, ***HLA-DQB1***, ***HLA-DRB1***, ***IL7R***, *KLRF1*, *LOC388796*, *MIR3201*, *MIR4759*, *PRF1*, *PSTK*, *PYHIN1*, *RAPH1*, *RNU7-13P*, *RNU7-63P*, *RNU7-64P*, *RNU7-69P*, *RNU7-6P*, *RNU7-7P*, *RNU7-85P*

Bolded texts represent genes known to be important to MDSC function

Ingenuity Pathway Analysis™ (IPA) software was utilized to predict downstream effects from this septic MDSC transcriptomic profile at 14 days after sepsis and revealed significant upregulation of genes involved in “growth of malignant tumor,” “proliferation of cancer cells,” and “inflammatory response” and downregulation of genes involved in “proliferation of lymphatic system cells” (Table 8).

**Table 8:** Predicted activation or deactivation of specific pathways based on MDSC mRNA expression from septic day 14 patients

Diseases or function	Predicted activation state *Z*-score
Inflammatory response*	2.187
Growth of malignant tumor*	2.607
Proliferation of cancer cells*	2.414
Proliferation of lymphatic system cells^#^	*− 2.351*

The top pathways and their corresponding *Z*-score within the functional category in severe sepsis/septic shock patients 14 days after insult

*The pathway is predicted to have significant activation

^#^The pathway is predicted to be downregulated

### microRNA prediction of target genes overlaps with significant genes found at day 14

Analysis using TargetScan revealed that the predicted gene targets of the differentially expressed miR from MDSCs isolated from septic patients at 14 days overlap with the genes found significantly expressed in septic patients vs healthy controls at day 14 (Table 9). Interestingly, the predicted gene targets for the downregulated miRs had a higher overlap with the genes upregulated at day 14. These also included genes associated with MDSC proliferation and MDSC immunosuppressive function from above: *CEACAM*, *FKBP5*, *HMGB2 LCN2*, *MMP8*, *MS4A4A*, *PFKFB3*, and *HLA-DQB1.*

**Table 9:** Day 14 septic MDSC miRs and their predicted gene targets

microRNA	Matched gene target
Downregulated	
hsa-miR-3175	*METTL7B*, *GPR84*, *LCN2*, *LTF*, *FKBP5*, *TMEM14E*, *UGCG*, *PAG1*, *FAIM3*, *ACVR1B*, *HES1*, *ABCA13*, *CLU*, *ARID5B*, *MS4A4A*, *CEACAM1*, *ELMO2*, *SLC36A1*, *PFKFB3*, *KIF1B*, *PFKFB2*, *MKI67*, *MAPK14*, *FBXO9*, *FBXW2*, *CHST7*, *GSR*, *FAM20A*, *CD63*
hsa-miR-4443	*PFKFB3*, *METTL7B*, *GCLM*, *KIF1B*, *MS4A4A*, *IL7R*, *CLU*, *ACVR1B*, *MAPK14*, *TDRD9*, *MMP8*, *SYNE1*, *SLC36A1*, *FKBP5*, *PLAC8*, *ABCA13*, *MKI67*, *LTF*, *FAM20A*
hsa-miR-3921	*PSTK*, *TDRD9*, *KLRF1*, *CEACAM1*, *FAIM3*, *ANKRD34B*, *KIF1B*, *CHST7*, *SLC36A1*, *ELMO2*, *PFKFB2*, *RAPH1*, *MAP 2 K6*, *IL7R*, *FKBP5*, *MARCH1*, *GCLM*, *C15orf37*, *METTL7B*, *C15ORF37*, *GCNT1*, *MKI67*, *PAG1*, *ENOSF1*, *FBXO9*, *GSR*, *MARC1*, *HLA-DRB1*, *FAM20A*
Upregulated	
hsa-miR-5000-5p	*PLAC8*, *TMEM14E*, *PSTK*, *ADAM9*, *CEACAM1*, *IL7R*, *ANKRD34B*, *ABCA13*, *HLA-DQB1*, *CD177*, *CHST7*, *GCLM*, *GPR141*, *FKBP5*, *MAP 2 K6*, *GCNT1*, *RAPH1*, *GSR*, *FAM20A*

List of select significant MDSC miRs from septic patients at day 14 and their predicted targets of significant genes of the same cells as analyzed by TargetScan software

## Discussion

Although post-sepsis immunopathology is complex, patients who exhibit CCI have persistent low-grade inflammation and immunosuppression that are known to contribute to their poor outcomes [19, 25]. Studies have shown that MDSCs play an important role in these phenomena [32, 44, 54]. Our research, along with others, has revealed that these cells, at least in regard to cell surface phenotype, are present in the blood of septic patients as soon as 24 h after sepsis onset and are persistent up to 42 days after the host’s initial infection in sepsis survivors with CCI. However, our current study has revealed that myeloid cells 4 days after sepsis, despite having MDSC phenotypic surface markers, are stimulatory toward T cells. This insight is important, as the timing of any immunomodulation after sepsis needs to consider if the host’s circulating immature myeloid cells do in fact require modification. Thus, personalized medicine for sepsis will require we understand the functional status of the host’s immature myeloid cells so that any patients receiving MDSC modification therapy at a specific time point would benefit from the therapy. In addition, since cellular epigenetics can be modified, microRNA expression presents itself as a potential modification therapy for plastic MDSCs in human sepsis [33].

Transcriptomic analysis of the MDSC genome at 14 days post-septic insult revealed a propensity for these cells to upregulate the inflammatory response with simultaneous downregulation of lymphatic cell proliferation. Additional analysis with IPA software revealed significant downregulation of the antigen presentation pathway (Additional file 2: Figure S2). Several studies implicate specific subsets of MDSCs as having unique roles in lymphocyte suppression. The expansion of the granulocytic subset of MDSCs is thought to be prevalent in sepsis [44]. Uhel et al., for example, was able to demonstrate that patients with early expansion of arginase-1 (*ARG1*)-producing G-MDSCs had strong correlations with increased T cell dysfunction and increased susceptibility to secondary infections in surgical intensive care unit (SICU) [20]. Although G-MDSCs were not the majority of circulating immature myeloid cells in the patients studied for this work, our data indicated that there was a gradual shift to the G-MDSC phenotype as the T lymphocyte proliferation suppressive function of MDSCs increased with time after surgical sepsis. Interestingly, a large portion of the isolated CD33^+^CD11b^+^HLA-DR^low/−^ did not meet the classic criteria of G- or M-MDSCs as defined in the cancer literature [55]. It is unclear if these CD33^+^CD11b^+^HLA-DR^low/−^CD14^−^CD15^−^ cells are the “early MDSC” phenotype described by Bronte [55] or immature myeloid cells still capable of appropriate differentiation. This subpopulation of isolated CD33^+^CD11b^+^HLA-DR^low/−^ that were non-granulocytic, non-monocytic in nature was not described in our previous work on human MDSCs in sepsis [20]. It should be noted that our current work included all septic patients while our previous publication on human MDSCs included only patients that met the Sepsis-2 criteria for severe sepsis and septic shock. In addition, there has been a dramatic reduction in “in-hospital” mortality due to the implementation of MEWS-SRS early warning systems for sepsis. These factors may have contributed to reduced total numbers of MDSCs and percentages of G-MDSCs observed in our previous publication [20]. Future studies will need to separately analyze these MDSC subtypes and will need to include some more novel cell surface markers. Additionally, the application of single-cell RNAseq and CITEseq techniques to these cells could be very beneficial to our understanding of post-sepsis dyscrasias, especially as myelopoiesis following certain inflammatory events is not only complex, but potentially unique to the disease process.

Many studies aim to target MDSC suppressive by-products, such as arginase-1, inducible nitric oxide synthase (iNOS), nitric oxide, and reactive oxygen species (ROS) [56]. Hübner et al. recently published a study showing that increased serum arginine breakdown in response to MDSC expansion caused dysfunctional T lymphocytes following cardiopulmonary bypass. However, dose-dependent l-arginine supplementation in vitro increased CD4^+^ and CD8^+^ proliferation and enhanced secretion of T cell-related cytokines, such as IFNγ, suggesting that arginine supplementation at specific time points after sepsis may warrant further investigation [57]. Preclinical mouse models have demonstrated that inhibitors of phosphodiesterase-5, sildenafil and tadalafil, downregulate iNOS and ARG1 activities, inhibiting MDSC function and leading to activation of antitumor host immunity and prolonged survival in mice [58–60]. Another clinical trial utilized tyrosine kinase inhibitors to target MDSCs in patients with renal carcinoma by blocking VEGF and c-kit signaling pathways. Patients receiving the tyrosine kinase inhibitors, sunitinib, had decreased levels of circulating MDSCs, reduced STAT3 activation and *ARG1* expression, and displayed elevated activity and proliferation of CD8^+^ cells [61]. Though the septic microenvironment is not as local as certain cancers, similar therapeutic strategies could be of interest.

Though there are several differences between the pathophysiology of cancer and sepsis, it is important to investigate a range of treatment options. In this study, we were able to define unique epigenetic expression patterns in MDSCs isolated at 4 vs 14 days following the host’s initial sepsis onset. Importantly, our work has also illustrated differences between those MDSCs collected from CCI and RAP patients at 14 days following initial sepsis onset. Our work also demonstrated an overlap between the predicted gene targets of the differentially expressed miRs and the differentially expressed genes from septic patients at day 14. Many of the significantly altered miRNAs detected, such as mir-21-5p, mir-181a, miR-106a, and mir-17-5p, are consistent with known regulators of differentiation and maturation of MDSCs in cancer and other autoimmune diseases. miRNA-21-5p expressed in myeloid cells is thought to be a negative regulator of the NF-κB pathway [62], is overexpressed in most tumor types [34, 63], and involved in myeloid progenitor expansion [34, 64, 65]. Our data revealed a significant upregulation of miR-21 at day 4 that shifted toward a significant downregulation by day 14 when MDSCs were potently immunosuppressive against T cell proliferation and capacity to produce cytokines. In septic murine models, in vitro blockade of miR-181 and miR-21 via antagomiR injection introduced a decrease in Gr1^+^CD11b^+^ cells, improved bacterial clearance, and reduced late-sepsis mortality by 74% [66, 67].

It is important to note that several of our miR expression results were not consistent with some of the previous literature (regarding up- vs downregulation), including human cancer results [33]. This illustrates potential heterogeneity in the myelopoietic response to specific diseases as well as the tissue/organ compartment in which the immature myeloid cell resides. It also reinforces the importance of understanding the temporal relation of myelopoiesis in the host after or during a disease process.

Though several of the current treatment options seem promising, our study has revealed the importance of proper timing in MDSC modification so that the targets of these therapies are indeed modifying functionally immunosuppressive MDSCs, rather than more broadly immature myeloid cells, potentially harming the patient. MDSCs may play a dual role during infection and sepsis. During early emergency myelopoiesis, MDSCs expand acutely and provide protection against microbial invasion by producing bactericidal molecules such as ROS and reactive nitrogen species (RNS) that counter the hyperinflammatory response [32]. However, the persistence of these cells can induce deleterious effects in the host.

Our current study was limited in several ways. Our study was restricted in its capacity to investigate a breadth of T lymphocyte produced cytokines, e.g., IL-4 and IL-33 were not in our current analysis. Additionally, we were limited in the ability to isolate MDSC subsets, specifically G-MDSCs, M-MDSCs, and early MDSCs, to investigate the unique suppressive effects of each subset as previous authors have shown. Future studies will need to elucidate the subset-specific mechanisms of MDSC immunosuppression. Currently, the literature only defines early MDSCs by the lack of granulocytic and monocytic specific lineage markers, making it difficult to completely characterize this cell population. However, the use of single-cell RNAseq/CITEseq to examine the transcriptomes of subset-specific human MDSCs after sepsis could help elucidate the properties of this early MDSC cell type. Also of note, there were some limitations of attrition over time. The analyses assumed that those missing data are missing at random and that may or may not be plausible. Lastly, we were unable to administer suggested therapeutics to potentially modify MDSC epigenetics, and thus their immunosuppression capacity.

## Conclusion

MDSCs contribute significantly to the host’s immunosuppressive status after human surgical sepsis. This effect is not immediate, but rather evolves over weeks, at which point circulating immature myeloid cells meet the classic criteria for MDSCs as defined in cancer patients. MDSC immunomodulation may well require multi-model therapy to improve septic patient outcomes. This multifaceted approach can potentially include epigenetic modifiers of these cells.

## Supplementary information

**Additional file 1:** **Figure S1.** T-cell Proliferation Titration Curves. Proliferation index titration curve of Septic T-cells (*n*=6) stimulated with 1:4, 1:2 and undiluted concentration of the soluble CD3/28 and cultured for 4 days at 37°C and 5% CO2. Unstimulated T cells served as control for these experiments.

**Additional file 2:** **Figure S2.** Differentially Expressed Genes from Patients 14 days after Sepsis involving the Antigen Presentation Pathway. Ingenuity Pathway Analysis illustration showing significant down regulation of many genes in the antigen presentation pathway. There are no significantly upregulated genes per the analysis. Orange = upregulation, blue = downregulation.

## Data Availability

The datasets used and analyzed during the current study are available from the corresponding author on reasonable request.

## Abbreviations

AML: Acute myeloid leukemia
ARG-1: Arginase-1
ATRA: All-trans retinoic acid
CCI: Chronic critical illness
COX2: Cyclooxygenase-2
CXCR: C-X-C motif chemokine receptor
E-MDSC: Early MDSC
G-MDSC: Granulocytic MDSC
ICU: Intensive care units
IL-2: Interleukin-2
iNOS: Nitric oxide synthase
LOS: Length of stay
LPS: Lipopolysaccharide
MDSC: Myeloid-derived suppressor cell
miR: microRNA
M-MDSC: Monocytic MDSC
PBMC: Peripheral blood mononuclear cells
PICS: Persistent inflammation, immunosuppression, and catabolism syndrome
RNS: Reactive nitrogen species
ROS: Reactive oxygen species
SICU: Surgical intensive care unit
SOFA: Sequential Organ Failure Assessment
TGF-β: Transforming growth factor-β
