# Supplementary figures and images for: Myeloid-derived suppressor cell function and epigenetic expression evolves over time after surgical sepsis
Source: Crit Care. 2019 Nov 13;23:355. doi: 10.1186/s13054-019-2628-x (PMC6854728; doi:10.1186/s13054-019-2628-x)

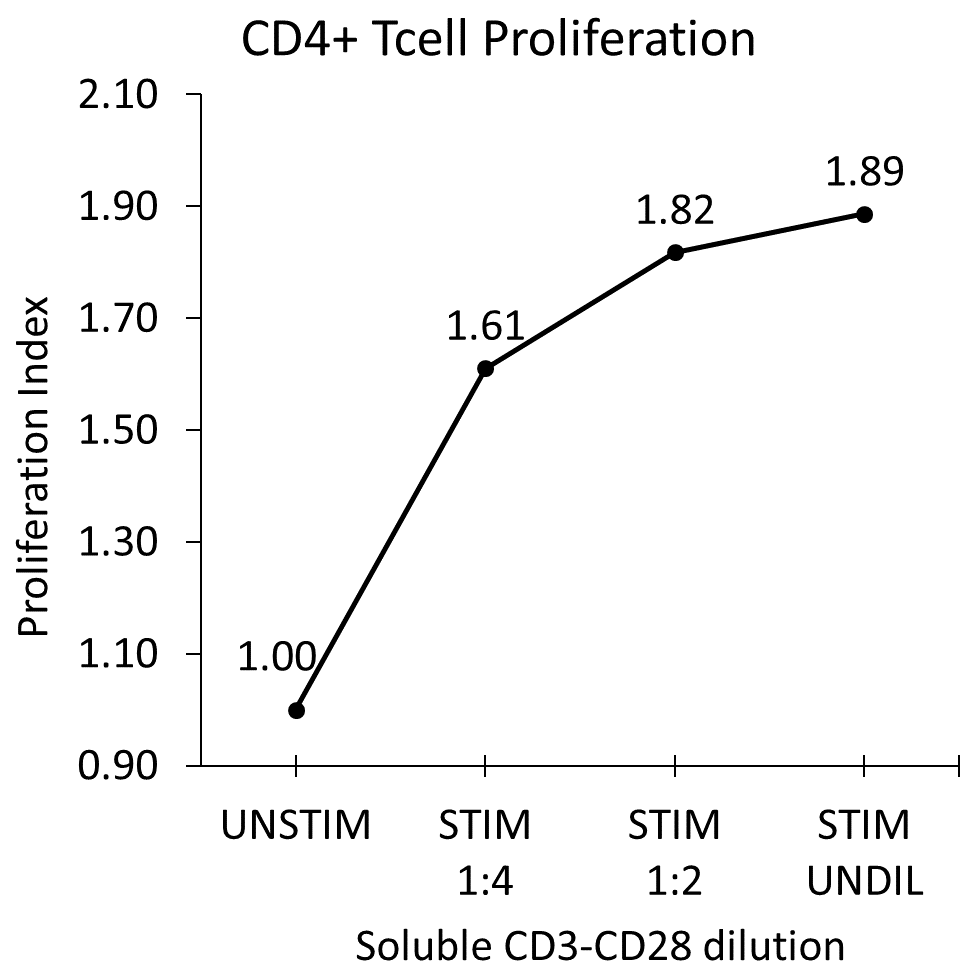


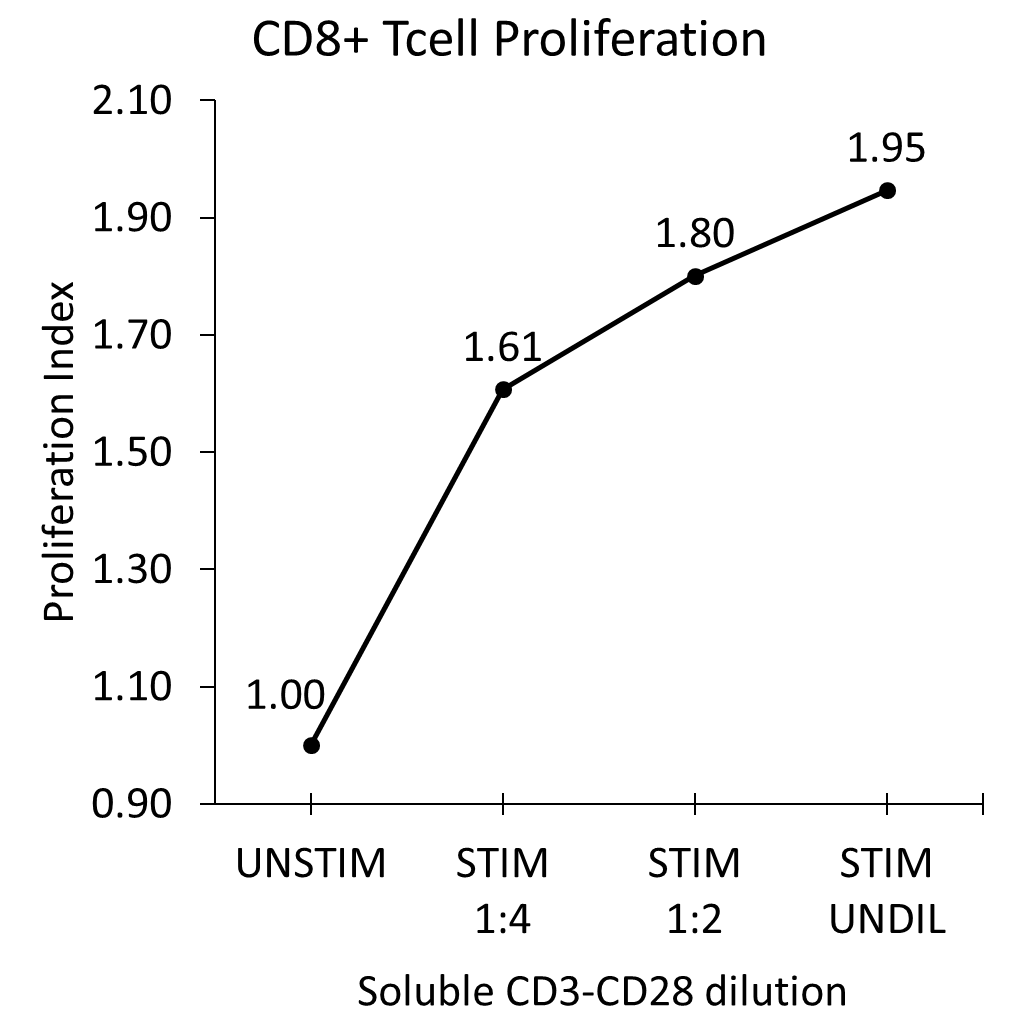

Supplement: Supplementary file 1 — Additional file 1: Figure S1. T-cell Proliferation Titration Curves. Proliferation index titration curve of Septic T-cells (n=6) stimulated with 1:4, 1:2 and undiluted concentration of the soluble CD3/28 and cultured for 4 days at 37°C and 5% CO2. Unstimulated T cells served as control for these experiments. [file 13054_2019_2628_MOESM1_ESM.docx]

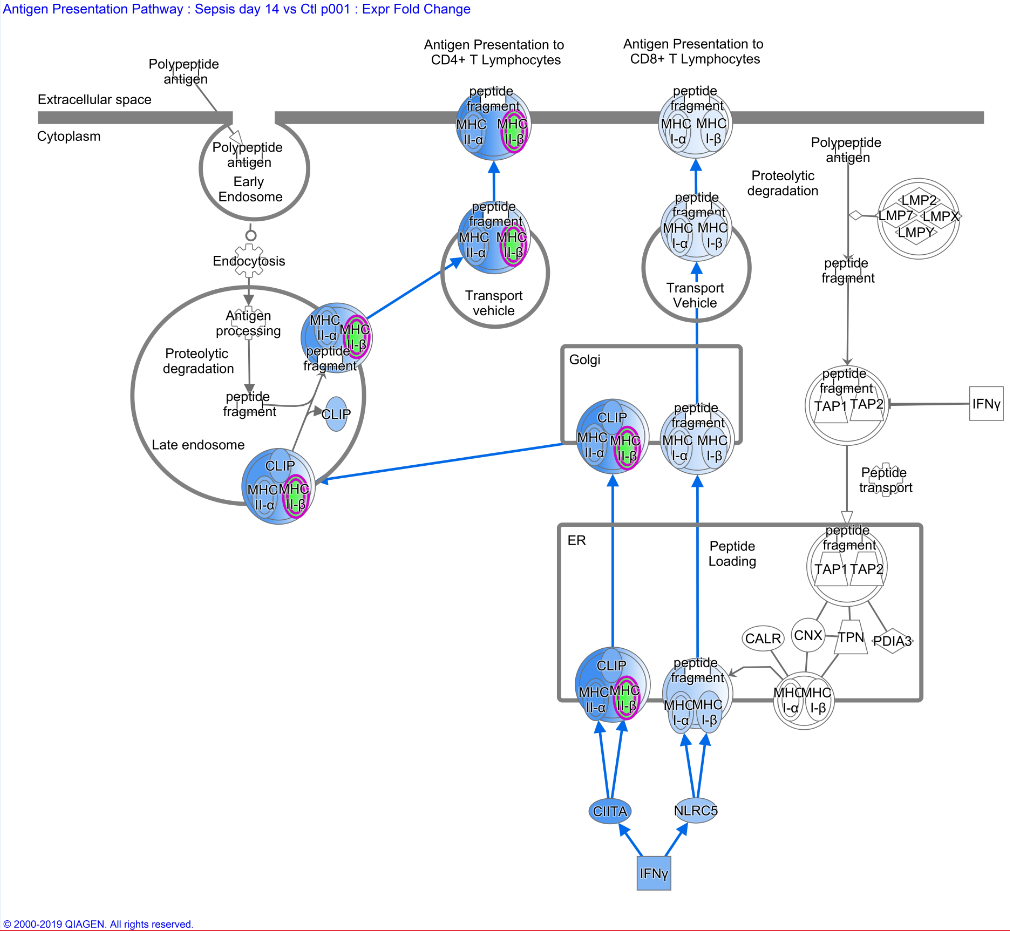

Supplement: Supplementary file 2 — Additional file 2: Figure S2. Differentially Expressed Genes from Patients 14 days after Sepsis involving the Antigen Presentation Pathway. Ingenuity Pathway Analysis illustration showing significant down regulation of many genes in the antigen presentation pathway. There are no significantly upregulated genes per the analysis. Orange = upregulation, blue = downregulation. [file 13054_2019_2628_MOESM2_ESM.docx]
